# Implementation of Instrumental Analytical Methods, Image Analysis and Chemometrics for the Comparative Evaluation of Citrus Fruit Peels

**DOI:** 10.3390/foods14234115

**Published:** 2025-12-01

**Authors:** Konstantinos Aouant, Paris Christodoulou, Thalia Tsiaka, Irini F. Strati, Dionisis Cavouras, Vassilia J. Sinanoglou

**Affiliations:** 1 Laboratory of Chemistry, Analysis & Design of Food Processes, Department of Food Science and Technology, University of West Attica, Agiou Spyridonos, 12243 Egaleo, Greeceestrati@uniwa.gr (I.F.S.); 2 Department of Biomedical Engineering, University of West Attica, Agiou Spyridonos, 12243 Egaleo, Greece

**Keywords:** citrus fruits, peel, by-product, image analysis, polyphenols, antioxidant and antiradical activity, ATR-FTIR spectroscopy, cross-block and multi-block analysis, network analysis

## Abstract

Citrus fruit cultivation and processing are constantly rising due to the increasing market demand and diverse utilization potentials. This generates large quantities of residues, predominantly composed of citrus peels. This study aimed to evaluate six different citrus peels using rapid and/or nondestructive instrumental analytical techniques such as ATR-FTIR spectroscopy, spectrophotometric assays, image textural analysis and physicochemical parameter determination. Image textural features managed to discriminate citrus peels based on their structure uniformity, which was found increased in lemon (*C. limon*) and yellow grapefruit (*C. paradisi*), whereas clementine (*C. clementina)* and red grapefruit (*C. paradisi)* images exhibited an increased non-uniformity of the structure. Physicochemical parameters provided insights into the quality characteristics of citrus peels, while their high ascorbic acid content seems to enhance their antioxidant activity. The obtained results from phenolic and flavonoid content determination indicated a high concentration of polyphenols in the peels, which is aligned with the ATR-FTIR spectra absorption bands. Furthermore, the spectrophotometric assays’ strong correlation suggests that the antioxidant activity of citrus peels is mainly attributed to polyphenols. Ultimately, a chemometric model was employed to provide a comprehensive understanding of the analytical methods’ interactions. Hence, citrus peels’ significant biochemical and, consequently, economic value can be highlighted, underscoring the importance of further research.

## 1. Introduction

The *Citrus* genus and the related genera *Fortunella*, *Poncirus*, *Eremocitrus* and *Microcitrus*, which belong to the Aurantioideae angiosperm subfamily of the Rutaceae family [1,2], consist of some of the most widely consumed and cultivated crops worldwide [3]. They are mainly grown in humid sub-tropical, semi-arid and Mediterranean climates [4], and the leading producing countries include China, Brazil, India, Mexico and Spain [5]. According to the Food and Agriculture Organization (FAO), citrus fruits are grown in more than 135 countries, with about 10.55 million hectares harvested and a global production of 166 million tons [5].

Citrus fruits are rich in both primary metabolites, such as sugars, vitamins and organic acids, which are essential for plant growth and provide necessary nutrients to humans [6,7], and secondary metabolites, often referred to as bioactive compounds, such as flavonoids, phenolic acids and carotenoids, which are produced in response to environmental stimuli and have potent health-promoting properties [7,8].

Extensive citrus cultivation and industrial processing are driven by the potential applications of their components in a wide range of industries, including food, cosmetic and pharmaceutical industries [9]. However, this leads to large quantities of residues annually that cause significant challenges and environmental impacts, such as high transportation costs, restricted access to water resources and the accumulation of organic waste in landfills [10]. In particular, citrus peels, which account for 40–55% of the fresh fruit mass, constitute the main by-product and contribute at least 10 million tons of waste annually worldwide [10,11], a considerable fraction of which is disposed of through burial or open burning [12]. Such practices not only contribute to environmental contamination but also result in the inefficient utilization of potentially valuable bioresources [12]. Citrus peels are a rich source of nutrients and bioactives, including vitamins, polyphenols and, most notably, essential oils, and therefore potent health-promoting effects can be attributed to these by-products [7,9].

The valorization of these inedible by-products through novel strategies [11] is facilitated in multiple ways, including EU directives on by-product management [13], the UN Sustainable Development Goals (SDGs) initiative targeting a 50% reduction in global food loss and waste by 2030 [11] and the growing industrial awareness of the economic and environmental impacts of proper waste management [14]. Collectively, these factors can foster sustainable practices and promote circular economy principles.

Considering the aforementioned, along with the important role of citrus fruits in the fruit industry, several studies [15,16,17,18,19,20,21,22,23,24,25] have been conducted on citrus fruit analysis. Most have focused primarily on the edible parts of citrus fruits [9] and typically either examine multiple species using fewer analytical methods or focus on fewer species with more extensive analyses. Therefore, this study focused on the combined implementation of various physicochemical, spectroscopic, image analysis and statistical methods to six different citrus peels in order to obtain the comprehensive information necessary for a thorough analysis and to provide new insights into their potential differences related to the physicochemical and quality characteristics, as these data are crucial for identifying further utilization opportunities for nutrient-rich by-products.

## 2. Materials and Methods

### 2.1. Sampling and Lyophilization

The samples were purchased from a local supermarket in Athens, Greece. Six different citrus fruits were analyzed: orange (*Citrus sinensis* cv. Valencia) cultivated in Lakonia, Greece; lemon (*Citrus limon*) cultivated in Aigio, Achaia, Greece; yellow and red grapefruit (*Citrus paradisi*) cultivated in Aigio and Crete (Greece), respectively; clementine (*Citrus clementina*) cultivated in Argolida, Greece; and kumquat (*Citrus margarita*) cultivated in South Africa. For each category of citrus fruit, 20–30 pieces were obtained and immediately transported to the laboratory, where they were placed under cold storage conditions at 4 °C. Peel (flavedo) samples were manually removed from 20 randomly selected individual citrus fruits for all the measurements. After the experiments were conducted on the fresh peels, they were stored at −80 °C for three days. Ten replicate samples were used for each experiment.

Frozen citrus peel samples were freeze-dried using a Gellert CryoDryer 20 lyophilizer (Langweid a. Lech, Bavaria, Germany) to reduce moisture content and obtain a consistent dry matrix for ATR-FTIR spectroscopy analysis, phenolics extraction and vitamin C determination, as the process preserves the bioactives and micronutrients [26,27]. The samples were freeze-dried once the thermocouples registered a temperature of −25 °C, and the vacuum was adjusted to 0.80 mbar pressure. The process was continued until the samples were totally dehydrated.

### 2.2. Image Analysis of the Surface of Citrus Peels

**Figure 1:**
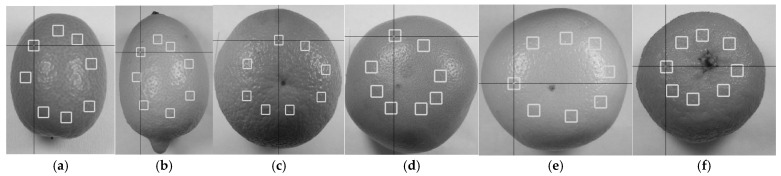
(**a**) Kumquat, (**b**) lemon, (**c**) orange, (**d**) red grapefruit, (**e**) yellow grapefruit and (**f**) clementine peel grayscale images used for textural feature calculation; ROI extraction indicated by the boxes.

The aim of this method was to evaluate the differences in the texture of citrus peels, following a methodology similar to Christodoulou et al. [28]. The samples were photographed using a Sony DSCW800/B (IXUS 100 IS) digital camera (Sony Europe Limited, Edinburgh, UK) with a lens aperture of *f* = 4.6 and a resolution of 1280 × 720 pixels, positioned 15 cm from the surface and under consistent lighting conditions. Fifteen (15) textural features were calculated by selecting a series of regions of interest (ROIs) (Figure 1) from the grayscale versions of each image, in order to estimate and quantify the differences in the textural properties among the samples. The first four (4) features were calculated from the statistical analysis of the first-order histogram of the grayscale image: mean intensity (the average intensity of all pixels of the image), standard deviation (STD) (deviation from the mean value), skewness (asymmetry of pixel intensities) and kurtosis (the degree of distribution in relation to the normal Gaussian curve). Six (6) features were calculated from second-order statistics of the grayscale image co-occurrence matrix: contrast (con) (changes in pixel intensity in an image), dissimilarity (dis) (alteration of image texture), energy (image uniformity), homogeneity (similarity of certain pixels in the image), correlation (correlation of gray levels) and angular second moment (ASM) (homogeneity in the image). Finally, five (5) features were calculated from the second-order statistics of the grayscale image run length table: short run emphasis (SRE) (the presence of consecutive rows of pixels with comparable intensities), long run emphasis (LRE) (longer consecutive series of pixels with similar intensity values), gray level non-uniformity (GLN) (variability in the distribution of gray levels), run length non-uniformity (RLN) (variations in the lengths of runs with similar intensity values) and run percentage (RP) (the distribution of runs) [29].

### 2.3. Physicochemical Measurements

The moisture content of the fresh and freeze-dried citrus peels was measured by placing 0.2–0.4 g of each sample on the pan of an electronic moisture analyzer (Kern MLS 50-3, KERN & SOHN GmbH, Balingen, Germany), whose internal halogen dryer heated the samples and induced moisture vaporization.

The measurements of total soluble solids (TSSs) of the fresh citrus peels were conducted with a hand-held refractometer (Kern Optics Analogue Brix Refractometer, ORA 80BB, KERN & SOHN GmbH, Balingen, Germany).

The ascorbic acid (vitamin C) content in the freeze-dried citrus peel samples was determined using the indophenol titration method, in which the acidified aqueous extracts were titrated with a solution of DCPIP (2,6-dichlorophenolindophenol) indicator dye [30]. The results for ascorbic acid were converted to a dry weight basis using the moisture content determined for the freeze-dried samples.

### 2.4. Extraction of Phenolic Compounds and Spectrophotometric Assays

For the preparation of the extracts, about 2 g of freeze-dried citrus peel sample was diluted in aqueous methanol (80% *v*/*v*) in a ratio of 1:10 (*w*/*v*). The solutions were stored in sealed flasks at 20 °C for 24 h, and the resulting diluted extracts were stored at 4 °C for further spectrophotometric analysis.

The total phenolic content (TPC) of freeze-dried citrus peel extracts was determined using a modified assay of the Folin–Ciocâlteu method proposed by Andreou et al. [31]. The results were expressed as mg of gallic acid equivalents (GAEs) per 1 g of citrus peel (DW). For the evaluation of the total flavonoid content (TFC), a modified micro-method of aluminum chloride (AlCl_3_) from Matić et al. [32] was employed. Measurements were performed at 415 nm. The results were expressed as mg of quercetin equivalents (QEs) per 1 g of citrus peel (DW), using standard solutions with a range of 5–500 mg·L^−1^ quercetin. To assess the antioxidant activity of freeze-dried citrus peel extracts, the Ferric Reducing Antioxidant Power (FRAP) assay was used according to the modified method of Lantzouraki et al. [33], and the obtained results were expressed as mg of Fe (II) per 1 g of citrus peel. The antiradical activity on the ABTS^●+^ radical of freeze-dried citrus peel extracts was determined according to the method described by Lantzouraki et al. [34] and was expressed as mg of Trolox Equivalents (TEs) per 1 g of citrus peel (DW). All spectrophotometric measurements were performed in triplicate with a Spectro 23, Digital Spectrophotometer (Labomed, Inc., Los Angeles, CA, USA). All of the above results were converted to a dry weight basis, using the moisture content that was determined for the freeze-dried samples.

### 2.5. Attenuated Total Reflectance–Fourier Transform Infrared Spectroscopy (ATR-FTIR)

ATR-FTIR analysis was conducted using an IRAffinity-1S FTIR Spectrometer manufactured by Shimadzu in Kyoto, Japan. The outer surface (flavedo) of the freeze-dried citrus peels was placed on the ATR surface and their spectrum was taken. Sample spectra and backgrounds were collected over a spectral range of 4000–499 cm^−1^, with an average of 20 scans at a resolution of 4 cm^−1^ and an ATR reference setting of 3284.77 cm^−1^. Data extraction, processing and analysis were performed using LabSolutions IR software (version 2.21) according to Ioannou et al. [35].

### 2.6. Statistical Analysis

The Python 3.10.6 scipy library (https://docs.scipy.org/doc/scipy/tutorial/, retrieved on 5 June 2025) was used to determine the textural features. The statistical analysis of citrus peel image features, physicochemical parameters, spectrophotometric results and ATR-FTIR spectroscopy data was performed using the non-parametric Kruskal–Wallis test and post hoc analysis.

### 2.7. Chemometric Analysis

The multiblock chemometric evaluation of the citrus peel datasets was designed following the conceptual framework mixOmics methodology, as implemented in the mixOmics R package (https://mixomics.org/ accessed on 5 September 2025).

Three independent analytical matrices were employed: an ATR-FTIR dataset consisting of absorbance values across characteristic wavenumbers, a spectrophotometric dataset comprising antioxidant and total phenolic parameters (Folin–Ciocâlteu, FRAP, ABTS^●+^, total flavonoids) and a physicochemical dataset including TSS (°Brix), moisture and vitamin C content. Each dataset contained identical sample indexing corresponding to six citrus peel classes (ten replicates per class). Prior to analysis, all numerical variables were auto-scaled by mean-centering and variance-scaling using the StandardScaler function of scikit-learn (https://scikit-learn.org/stable/ accessed on 5 September 2025), ensuring that all features contributed equally regardless of their original magnitude or units.

Exploratory multivariate evaluation and supervised classification were conducted in an integrated manner, and we performed Linear Discriminant Analysis (LDA) for each analytical block, as well as for the concatenated fusion through combinations of two or all three blocks, enabling the discrimination of the six citrus peel classes within a unified two-dimensional space. LDA was chosen to explore relationships between the datasets by finding linear combinations (linear discriminant variates) that maximize their correlation. Model performance was evaluated by five-fold stratified cross-validation, and the mean classification accuracy together with the standard deviation was reported. Furthermore, data fusion was implemented by horizontally concatenating the three auto-scaled matrices into a single multiblock dataset.

Pairwise Pearson correlation coefficients were then computed among all variables across the “combinations of two” data space. To further explore pairwise block relationships, cross-correlation heatmaps were generated for all block combinations: ATR-FTIR versus spectrophotometric, ATR-FTIR versus physicochemical and spectrophotometric versus physicochemical. Each heatmap depicted the Pearson correlation matrix between the variables of the two respective blocks, colored with a diverging “blue–red” scale centered at zero. The top five variable pairs with the highest absolute correlation values were listed in a table below each heatmap, providing clear examples of cross-domain feature dependencies.

Also, a correlation network was constructed, taking into consideration the fused data space in which each node represented a variable and each edge connected variables exhibiting an absolute correlation coefficient (|r|) ≥ 0.60. Nodes were color-coded according to their analytical origin (blue for ATR-FTIR, green for spectrophotometric and red for physicochemical variables), while the strongest associations (|r| ≥ 0.80) were emphasized with bold edges to highlight potential inter-block interactions. The network layout was generated using the Fruchterman–Reingold spring algorithm implemented in NetworkX (https://networkx.org/ accessed of 5 September 2025), and feature labels were standardized for readability by removing analytical prefixes.

All chemometric analyses were executed in Python (v3.10.6) using the pandas (https://pandas.pydata.org/ accessed on 5 September 2025), numpy (https://numpy.org/ accessed on 5 September 2025), scikit-learn (https://scikit-learn.org/stable/ accessed on 5 September 2025), matplotlib (https://matplotlib.org/ accessed on 5 September 2025), seaborn (https://seaborn.pydata.org/ accessed on 5 September 2025) and network (https://networkx.org/ accessed of 5 September 2025) libraries. Despite being implemented in Python, the analytical logic and multiblock integration strategy strictly followed the principles of DIABLO as formulated in mixOmics (R), ensuring methodological consistency with state-of-the-art data fusion approaches commonly employed in metabolomics and spectroscopic data integration.

## 3. Results and Discussion

### 3.1. Textural Image Analysis of Citrus Fruit Peels

**Figure 2:**
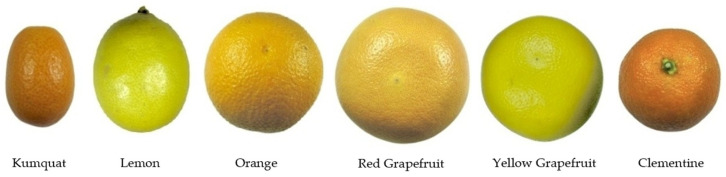
Representative photos of the citrus fruit peels studied (photographs were taken by Konstantinos Aouant, 2025).

**Figure 3:**
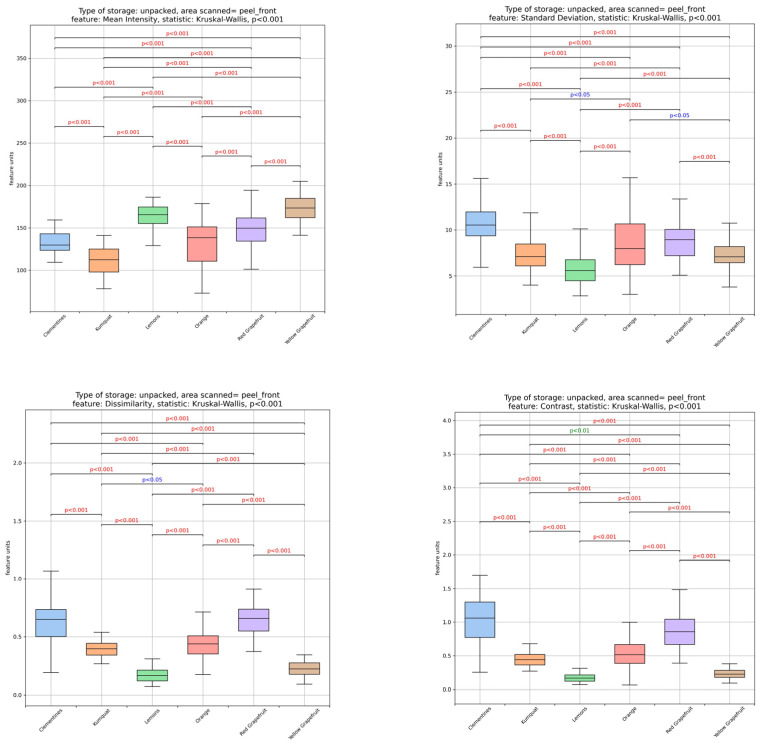

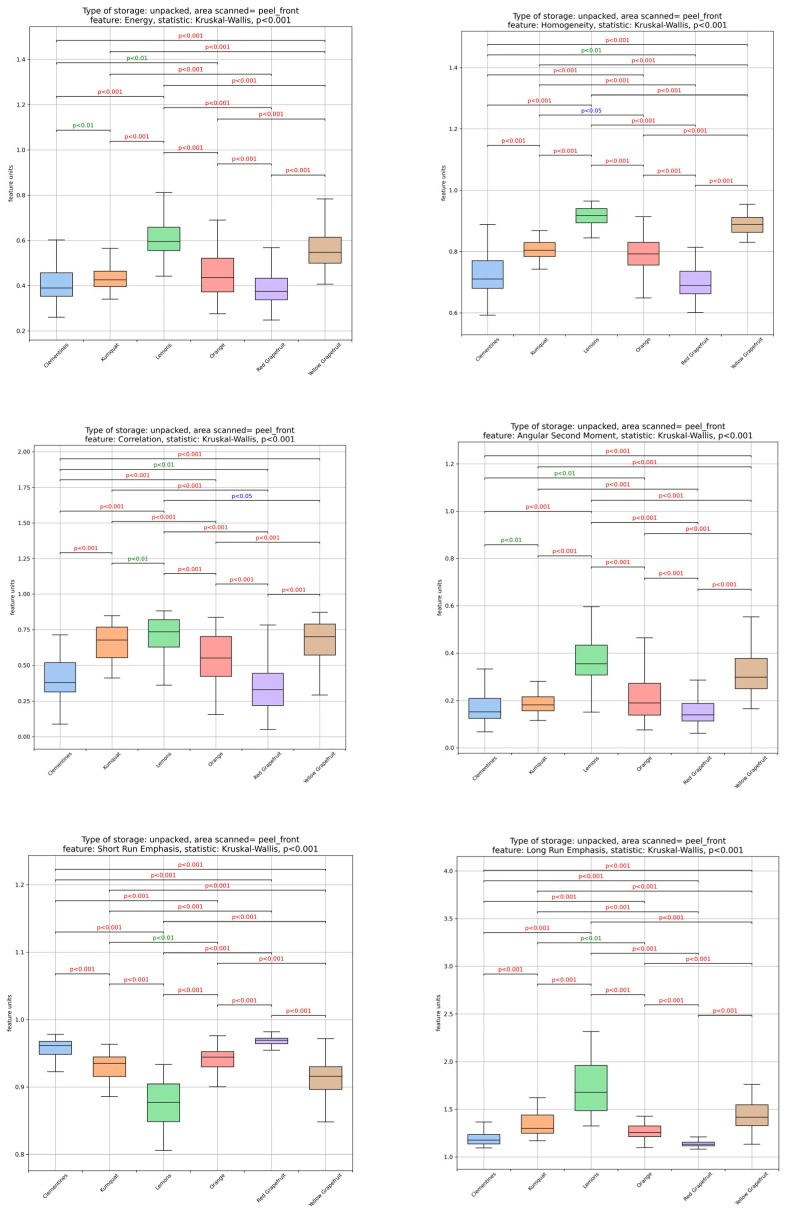

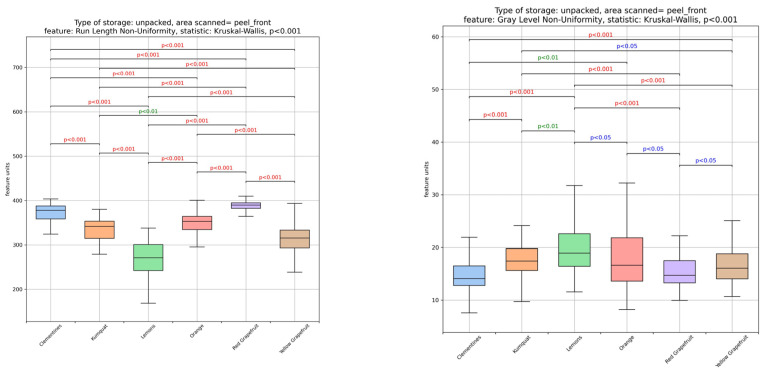
Fluctuations and *p*-values in image analysis-derived features [mean intensity, standard deviation, dissimilarity, contrast, energy, homogeneity, correlation, angular second moment (ASM), short run emphasis (SRE), long run emphasis (LRE), run length non-uniformity (RLN) and gray level non-uniformity (GLN)] of the citrus peels. (blue—clementine peels, orange—kumquat peels, green—lemon peels, red—orange peels, purple—red grapefruit peels, brown—yellow grapefruit peels).

An image analysis was used to extract information about texture variation in the peels of the citrus fruits studied. Figure 2 illustrates the differences in peel appearance among citrus fruits. More specifically, the texture of the peels was evaluated based on textural features (see Figure 3) derived from their grayscale images, which were processed using image analysis and machine learning methods.

The results showed that the structure of lemon and yellow grapefruit peels was the most uniform. Orange and kumquat peels demonstrated intermediate levels of uniformity, whereas clementine and red grapefruit peels were less uniform in the grayscale images.

All the features based on the second-order statistics of the grayscale images in the co-occurrence matrix seem to confirm the aforementioned. In particular, the homogeneity of lemon and yellow grapefruit peels was significantly (*p* < 0.05) higher, confirming their greater structural uniformity compared to other citrus peels. Additionally, the higher (*p* < 0.05) values of the correlation, angular second moment and energy features confirmed the increased uniformity of the structure of lemon and yellow grapefruit peels. Meanwhile, clementine and red grapefruit peels exhibited the lowest (*p* < 0.05) values for these features, indicating an increased structural non-uniformity. Conversely, the increased uniformity of lemon and yellow grapefruit peels, as indicated by the lower (*p* < 0.05) contrast and dissimilarity values, was confirmed when compared to clementine and red grapefruit peels. The values of all the textural features previously mentioned for orange and kumquat peels were intermediate. Similar results were obtained from features derived from the second-order statistics of the run length matrix of the grayscale images. Lower (*p* < 0.05) short run emphasis (SRE) and run length non-uniformity (RLN) values, alongside higher (*p* < 0.05) long run emphasis (LRE) values, confirmed the uniform structure of lemon and yellow grapefruit peels. Once again, orange and kumquat peels exhibited mid-range values, while the increased non-uniformity of red grapefruit and clementine peels was reaffirmed. Ultimately, the mean intensity and standard deviation (STD) values, which were calculated based on the first-order statistics of the grayscale images’ histograms, also support the previously discussed observations.

According to Sinanoglou et al. [36], increases in standard deviation, contrast, dissimilarity, short-run emphasis and run length non-uniformity values indicate an uneven distribution of gray levels in image structures, resulting in the increased gray level inequality and non-uniformity of structures in images. This confirms the non-uniformity of the structures in images of red grapefruit and clementine peels.

The quality of citrus fruits is directly affected by climatic conditions [37]. Unlike the other species examined, lemon trees tend to grow, flower and bear fruit year-round [38]. This could potentially explain the uniformity observed in lemon peels, as it suggests that the species is adaptable throughout the year. Furthermore, the similarities observed in the texture of the peels of lemons and yellow grapefruits could be related to their shared geographical origin, Aigio, Achaia, Greece, as factors such as climate, soil quality (pH, organic matter and nutrient content), humidity, temperature and sun exposure play a significant role in citrus cultivation and the quality of the fruit [37]. The uniformity of lemon and yellow grapefruit peels may also be related to the carotenoid content of the peels and the fruit’s tolerance to chilling injury (CI). Previous studies have shown that citrus fruits with lighter-colored peels that accumulate low levels of carotenoids are more susceptible to CI [39]. Specifically, Rey et al. [39] report that CI symptoms on red grapefruit peels were confined to the lighter yellow areas, whereas regions with intense red coloration due to lycopene accumulation showed no symptoms. This observation suggests a potential correlation between high carotenoid content and the non-uniformity of citrus peels, as evidenced by the quantification of texture features derived from image analysis.

Although image analysis is one of the most interesting modern non-destructive techniques [40], its application remains somewhat limited. Some studies use image analysis to categorize diseases or defects that appear on the peels of citrus fruits [41,42]. Of particular interest are the studies by Nuño-Maganda et al. [43], who developed a machine vision system for monitoring and sorting citrus fruits based on color and size, and by Qadri et al. [44], who investigated the use of machine vision to classify eight varieties of citrus fruit by photographing their leaves. Digital image processing has also been used to classify and determine the shelf life of fruits and vegetables, as well as predict their quality during storage [28,36,45,46,47].

### 3.2. Physicochemical Parameters of Citrus Fruit Peels

The physicochemical parameters, including the moisture content (%), ascorbic acid content (mg/100 g DW peel) and total soluble solids (°Brix), of citrus peel samples were evaluated. The results of these analyses are presented in Table 1.

According to our findings regarding moisture content, the kumquat peel had the highest (not necessarily significant) moisture content value at 74.95%, while the orange peel had the lowest value at 63.92%. The findings of the present study regarding red and yellow grapefruit are consistent with those of Czech et al. [20]. Furthermore, Gómez-Mejía et al. [48] reported a higher moisture content in clementine peels than in lemon peels; Pathak et al. [49] observed a higher moisture content in orange peels than in lemon peels; and Souza et al. [19] confirmed the moisture content in kumquat peels. However, the moisture content of citrus peels can vary for several reasons, including cultivar, cultivation method, harvest season, ripening stage, climate and soil conditions [50,51]. This is evident when comparing our results with those of Barros et al. [15], who found the moisture content of Pera and Lima orange peels to be 66.6% and 70.3%, respectively. These values differ slightly from those of the present study, in which the moisture content of Valencia orange peels was found to be 63.92%. It is important to note that the high moisture content of citrus peels creates favorable conditions for biochemical and microbiological degradation, such as mold growth [52,53]. Additionally, since citrus peels are typically processed using treatments such as dehydration to ensure stability [53], it is crucial to evaluate the initial moisture content of the fresh peel [54].

The ascorbic acid content was found to be significantly higher (*p* < 0.05) in the peels of yellow and red grapefruits, followed by orange, kumquat, clementine and lemon peels, which did not present any statistically significant differences among them. Our results align with those of Sir Elkhatim et al. [16] and Fatin Najwa and Azrina [55], who also observed significantly higher (*p* < 0.05) ascorbic acid concentrations in grapefruit peels than in orange and lemon peels. Furthermore, Alós et al. [56] reported lower (*p* < 0.05) ascorbic acid values in clementine peels than in orange and grapefruit peels. However, our findings regarding the ascorbic acid content of kumquat peels align with those of Pawełczyk et al. [57]. The factors affecting vitamin C levels in different citrus fruits include climatic and environmental conditions, the position of the fruit on the tree, fruit exposure to light, post-harvest handling and storage, the species and variety of citrus fruit and the stage of ripeness at harvest [15,39,55].

Regarding TSS, the °Brix values, which represent the concentration of dissolved solids, showed that kumquat and orange peels had the highest total soluble solids content. The °Brix values of the other citrus peels did not show a significant difference. In line with our findings, Souza et al. [19] reported that the total soluble solids of kumquats were 16.41°Brix. In contrast, Pham et al. [50] found that the °Brix value of lemon peels was significantly lower, at 2°Brix. This discrepancy may be due to differences in the fruit variety, cultivation method, harvest season and stage of ripeness [51,58]. According to the literature, the determination of total soluble solids in citrus fruits focuses mainly on the juice, since the pulp and juice are the edible parts of the fruit [15,59,60,61,62]. There are few references to the peel of the fruit. Interestingly, Rivas-Cantu et al. [63] mentioned in their research a higher content of soluble solids (mainly fructose, glucose and sucrose) in orange peels compared to the fruit pulp.

### 3.3. Spectrophotometric Assays of Citrus Fruit Peels

The total phenolic and flavonoid content, as well as the antiradical and antioxidant activity, of citrus fruit peels (on a dry weight basis) were quantified using spectrophotometric methods. The results are displayed in Table 2.

A significantly (*p* < 0.05) higher concentration of phenolic compounds was revealed in the peels of yellow and red grapefruit, whereas kumquat peels exhibited the lowest (*p* < 0.05) total phenolic content. These findings are consistent with those of Sir Elkhatim et al. [16], who reported that grapefruit peels contained the highest content of phenolic compounds, followed by lemon and orange peels. Similar observations were also reported by Li et al. [64], Mehmood et al. [65] and Zapata et al. [66]. Furthermore, Ramful et al. [67] conducted a comprehensive analysis of twenty-one varieties of citrus peel extracts and found that orange peels had the highest phenolic content, followed by clementine and kumquat peels. However, Diab [68] reported that lemon peels had a phenolic content 2.3 times higher than grapefruit peels, and Goulas and Manganaris [69] stated that Valencia orange peels had a higher phenolic content than grapefruit peels from various cultivars.

Citrus peels are widely recognized as a rich source of flavonoids, with a higher flavonoid content than the edible part of the fruit [24,69,70,71]. The results of the present study indicated that lemon peels exhibited significantly higher TFC values (*p* < 0.05), at 31.90 mg QE/g DW, followed by yellow grapefruit, red grapefruit and orange peels. Clementine and kumquat peels showed significantly lower TFC values (*p* < 0.05). These results are consistent with those of Mare et al. [72] and Singh and Immanuel [73], who reported a higher TFC in lemon peels than in orange peels. They are also consistent with the findings of Chen et al. [74], who observed a higher TFC in orange peels than in kumquat peels. Furthermore, in agreement with the findings of this study, Diab [68] reported that lemon peels had a higher flavonoid content than grapefruit peels. Similarly, Wang et al. [75] found that lemon peels had a higher flavonoid content than orange, clementine and kumquat peels. Findings that differ from those of the present study were reported by Sir Elkhatim et al. [16], who observed that the flavonoid content of orange and grapefruit peels was significantly higher than that of lemon peels. Additionally, Goulas and Manganaris [69] reported higher flavonoid concentrations in orange peels than in various grapefruit cultivars. Ramful et al. [67], meanwhile, observed that orange peels had a higher flavonoid content than lemon and kumquat peels. According to a literature review, results differing from those of the present study regarding TFC and TPC can be attributed to genetic and environmental factors (e.g., cultivation practices, light and temperature exposure, harvest period), as well as experimental approaches (e.g., sample pretreatment, extraction methods, reference standards) [16,17,24,68,71,76].

Citrus peels are a rich source of numerous antioxidant compounds, including ascorbic acid, carotenoids and polyphenols [77]. It is well established that, among these bioactive compounds, polyphenols, and particularly flavonoids, play a key role in contributing to the antioxidant capacity of citrus peels [16,21,24,73]. Evaluating the antioxidant and antiradical activities of citrus peel samples using the FRAP and ABTS^•+^ spectrophotometric methods, respectively, revealed that kumquat peels exhibited significantly lower values for both assays (*p* < 0.05). Conversely, lemon and yellow grapefruit peels exhibited significantly (*p* < 0.05) higher antioxidant and antiradical activities, respectively. Consistent with the results of the present study, Bratovcic et al. [78] and Singh and Immanuel [73] confirmed the higher antioxidant and antiradical activity of lemon peels compared to orange peels, whereas Diab [68] reported that lemon peels exhibited a higher antioxidant activity than grapefruit peels. Some variations observed compared to the previous literature, such as in the determination of TPC and TFC, could be attributed to different methodological approaches and differences in sample characteristics (e.g., cultivar, maturity and harvesting time) [76,79].

Very strong or high positive Pearson correlations were found between all pair combinations of total phenolic content (TPC), total flavonoid content (TFC), antiradical activity and antioxidant activity of citrus fruit peels (Table 3). This indicates that the strong correlations observed in the spectrophotometric assays in this study confirm that the total phenolic content is directly related to both the flavonoid content and antioxidant activity. Therefore, flavonoids are the main contributors to the antioxidant activity of citrus peels. The lower correlation values observed for TPC and TFC with antiradical activity could be attributed to the diverse antioxidant mechanisms, which could limit the ability of the phenolic compounds and flavonoids found in citrus peel extracts to scavenge radicals [21].

Collectively, the spectrophotometric assays reveal a consistent pattern in which *C. paradisi* (red and yellow grapefruit) and *C. limon* (lemon) peels exhibit a high antioxidant activity. This aligns with their high ascorbic acid content, as observed in the physicochemical results, suggesting a combined antioxidant contribution from both polyphenols and ascorbic acid.

### 3.4. Interpretation of Attenuated Total Reflection–Fourier Transform Infrared (ATR-FTIR) Spectra

**Figure 4:**
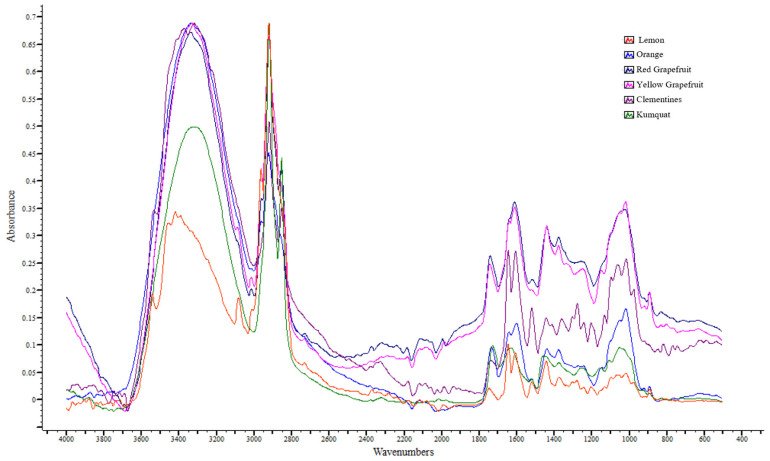
ATR-FTIR spectral overlay of citrus peels.

The ATR-FTIR spectra of freeze-dried citrus peel samples (Table 4, Figure 4) were interpreted based on the previously published literature data to ensure a detailed identification of the absorption regions. The bands were obtained within the spectral range of 4000–499 cm^−1^, with all spectra presenting absorption bands typically associated with phenolic and aromatic compounds, lipids and carbohydrates.

Specifically, the absorption band at 3630–3410 cm^−1^ is appointed to O-H stretching vibrations in pectin and phenolic compounds [80,81,82,83], as well as to the hydrogen-bonded hydroxyl groups of polysaccharides (hemicellulose and cellulose) or polyphenols [84]. The band at 3300 cm^−1^ is related to the stretching vibrations of the hydroxyl groups present in water, carbohydrates (pectin and cellulose), organic acids (ascorbic and citric acid) and polyphenols (flavonoids) [82,85,86]. The peaks at 2962–2922 cm^−1^ and at 2850 cm^−1^ indicate the presence of asymmetric and symmetric C(sp^3^)-H stretching vibrations in methylene and methyl groups of carbohydrate and carboxylic acids [84,87,88]. Hădărugă et al. [89] also mention similar bands appearing in flavonoid glycosides. The band at 1730 cm^−1^ is ascribed to C=O stretching vibrations of organic acids and ester functional groups [46,80]. More precisely, these vibrations could be associated with aromatic compounds of fruits, such as ethyl hexanoate and methyl and ethyl butanoate [90], as well as with cutin, a waxy polymer composed from esterified hydroxy fatty acids that is a major component of plant cuticles and phenolic esters [47,81,91]. The absorption band at 1643 cm^−1^ can be attributed to both bending vibrations due to the deformation movements of the hydroxyl groups of polyphenols, carbohydrates, organic acids and water [85,90] and to the C=O asymmetric stretching vibration of flavonoids [89].

The strong peak at 1600 cm^−1^ suggests the C-C stretching vibration of pectin and aromatic compounds [91,92] and the asymmetric stretching vibrations of the carboxylic group of carbohydrates, particularly of pectin [83,93]. The 1517 cm^−1^ band corresponded to C=C-C stretching vibrations in the aromatic ring of phenolic compounds [81,87]. The peak in the range of 1439–1400 cm^−1^ suggests the COOH symmetric stretching vibration of the pectin structure, and it also could be assigned to the combination of O-H bending and C-H rocking in monosaccharides, respectively [46,83]. The band at 1370–1360 cm^−1^ is associated with the C-O stretching vibration of the carboxylic group related to organic acids and carbohydrates [84], and the bands at 1330 and 1300 cm^−1^ are attributed to the CH bending vibration of polysaccharides and the cellulose ring [93]. The bands in the range of 1280–1274 cm^−1^ are assigned to the O-H bending vibrations of cutin and polysaccharides [91], as well as to the amide III band, due to the combined C-N stretching and N-H and O=C-–N bending vibrations in proteins [91,93]. The band at 1240 cm^−1^ corresponds to the C-O stretching vibrations of polyphenols and carbohydrates [36] and to the C=C stretching vibrations of aromatic polyphenols [84]. The peak in the range 1182–1090 cm^−1^ is related to the polysaccharide content of citrus peels. Specifically, the band at 1182 cm^−1^ is ascribed to the C-C stretching vibrations of cellulose [84] and also to the asymmetric C-O-C bridge stretching of cellulose [94]. The band at 1147 cm^−1^ is assigned to the C-O-C stretching vibrations of cutin as well as the glycosidic bond of pectin and cellulose [91,95], and the band at 1100–1090 cm^−1^ is attributed to the C-O και C-C stretching vibrations of pectin and polysaccharides [96]. The band at 1055 cm^−1^ is related to C-O bending vibrations and C-OH stretching vibrations in the structure of carbohydrates (cellulose, sucrose) [36,94,97]. The strong peak at 1016–1012 cm^−1^ suggests the C-O and C-C stretching vibrations in pectin and cellulose [91], the C-OH bending vibrations in carboxylic acids, alcohols and carbohydrates (e.g., cellulose) and the O-CH bending vibrations of pectin and other polysaccharides [88,91,93]. According to Küçük [88], these peaks confirmed the cellulosic structure of *Citrus paradisi* peels.

The absorptions at 975 and 738 cm^−1^ are associated with the *trans*- and *cis*-C-H out-of-plane bending vibrations of the carotenoids, respectively [98]. The band at 920 cm^−1^ is related to the C=C out-of-plane bending vibrations of alkenes [86] and to the C-H bending vibrations of the benzene ring of phenols [84]. The absorptions in the range of 890–840 cm^−1^ are assigned to the C-H bending vibrations of 1,4-disubstitution (para) in aromatic rings [81], and the band at 812 cm^−1^ is also assigned to the C-H group vibrations of phenolics aromatic rings [99]. The band at 765 cm^−1^ is related to twisting bending vibrations of the C-OH group [89] and to the C-H out-of-plane bending vibrations of the ortho-substituted aromatic rings [36]. The band at 623 cm^−1^ is assigned to the O-H out-of-plane bending vibrations of pectin [36], and the band at 586 cm^−1^ is associated with the out-of-plane C-H bending vibrations of polyphenols and flavonoids [92,100]. Lastly, the range of 530–524 cm^−1^ is related with C-O-C in-plane bending vibrations in the glycosidic bond of pectin [36].

Interpreting the ATR-FTIR spectra (Table 5) of the citrus peels produced the following results. In particular, the absorption band at 3300 cm^−1^ displayed a statistically significant (*p* < 0.05) increased intensity in the kumquat and orange peels, which can be attributed to their high water, carbohydrates and ascorbic and citric acids compared to the other samples, particularly in orange peels. The absorption observed in the other citrus peel samples could be attributed to their higher phenolic content, which contributes to their antioxidant activity, as confirmed by spectrophotometric assays. Strong peaks at 2962–2922 cm^−1^ and 2850 cm^−1^ have been reported in numerous studies on citrus peels [23,84,88,89], as they are rich in volatile compounds (e.g., D-limonene, α-pinene, linalool and various monoterpenes) containing methyl groups. These compounds are the main components of essential oils, which are present in large quantities in citrus peels [10]. The strong bands at 1600 cm^−1^, 1517 cm^−1^, 1370–1360 cm^−1^ and 1240 cm^−1^, along with the peaks in the range of 890–765 cm^−1^, confirm the high phenolic and flavonoid content of citrus peels, in accordance with the results obtained from spectrophotometric assays. Lastly, the intensity of the peak at 1016–1012 cm^−1^ indicates a high concentration of polysaccharides (e.g., pectin and cellulose) in citrus peels, which aligns with previous studies [80,83,101,102].

### 3.5. Chemometric Analysis of the Analytical Methods

Three levels of chemometric modeling approaches, single-block, cross-block and multi-block, were developed as described in Section 2.7 to integrate the ATR-FTIR, spectrophotometric and physicochemical datasets, aiming to reveal comprehensive biochemical patterns from the distinct analytical results and to evaluate their individual and synergistic discriminatory efficiency among citrus peels.

#### 3.5.1. Single-Block Model

**Figure 5:**
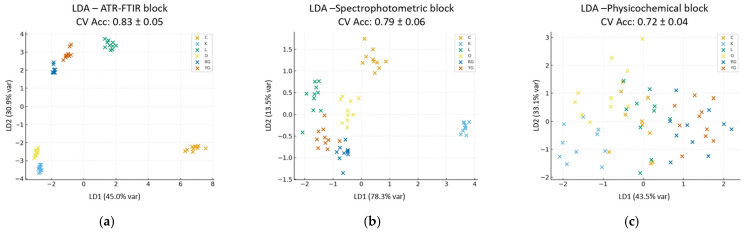
Single-block score plots: (**a**) ATR-FTIR block; (**b**) spectrophotometric block; (**c**) physicochemical block.

The single-block score plots (Figure 5) reveal a moderately distinct sample distribution across the two linear discriminants (LD1, LD2). In particular, the ATR-FTIR block (Figure 5a) exhibits the strongest class separation (44.98% and 30.85% of explained variance for linear discriminants 1 and 2, respectively), whereas the spectrophotometric (Figure 5b) and physicochemical (Figure 5c) blocks display weaker and overlapping sample clusters, suggesting a lower discriminative power when considered individually.

As shown in Figure 5, this information, when considered independently, is insufficient for complete class discrimination. Additionally, the diffused distribution observed in the physicochemical block suggests that, while these features contribute general compositional information, they are not sufficiently distinctive for strong classification on their own, as they vary only slightly among the samples. Nonetheless, although spectrophotometric and physicochemical blocks are individually less discriminant, they remain valuable, as they capture complementary aspects of the samples’ biochemical and compositional variability, which can be exploited more effectively through data fusion analysis.

#### 3.5.2. Cross-Block Fusion

**Figure 6:**
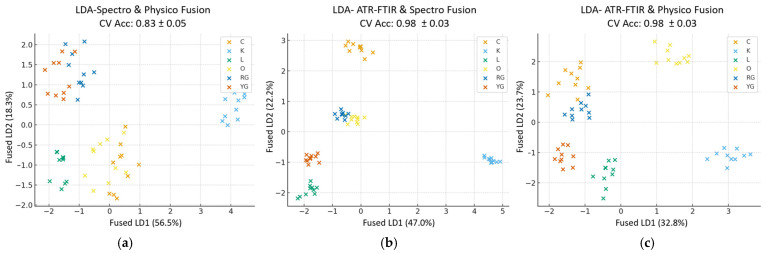
Cross-block score plots: (**a**) physicochemical and spectrophotometric fused score plot; (**b**) ATR-FTIR and spectrophotometric fused block; (**c**) ATR-FTIR and physicochemical fused block.

As an initial step toward data fusion, cross-block models were developed by combining two blocks in each model to assess their joint contribution to sample discrimination. The resulting score plots (Figure 6) provide insights into the interaction between datasets and their contribution to sample discrimination.

The fused score plot of spectrophotometric and physicochemical blocks (Figure 6a) exhibited a moderate discrimination, although a notable overlap remained among some citrus peel classes, suggesting that the integration of these datasets represents mainly general compositional variation rather than distinct structural differences. However, the classification accuracy (0.83 ± 0.05) indicates a significant improvement, especially over the physicochemical block, and demonstrates that their fusion enhances class discrimination. Although it did not exceed the strong discriminative ability of the spectrophotometric block, the fusion produced a more stable and interpretable model, capturing complementary variability related to both antioxidant composition and physicochemical traits.

In contrast, both the ATR-FTIR and spectrophotometric (Figure 6b) and the ATR-FTIR and physicochemical (Figure 6c) fused score plots displayed a high class discrimination (classification accuracy: 0.98 ± 0.03), indicating that the ATR-FTIR spectra provide the primary discriminant information, while the secondary datasets enhance the model efficiency by capturing broader compositional variations. Particularly, the ATR-FTIR and spectrophotometric fused score plot, showing a tight grouping of samples, indicates a strong synergy between the molecular vibrations provided by ATR-FTIR spectra and the antioxidant compounds of the samples, resulting in well-discriminated classes and confirming that integrating ATR-FTIR and spectrophotometric data yields the best classification performance. The ATR-FTIR and physicochemical fused score plot, which also provides an excellent class discrimination with minimal overlap, seems to benefit mainly from ATR-FTIR spectra bands, while the physicochemical data seem to reinforce rather than drive class discrimination, providing complementary information. The equivalent accuracy of these two fused score plots confirms the key role of the ATR-FTIR block in sample discrimination.

**Figure 7:**
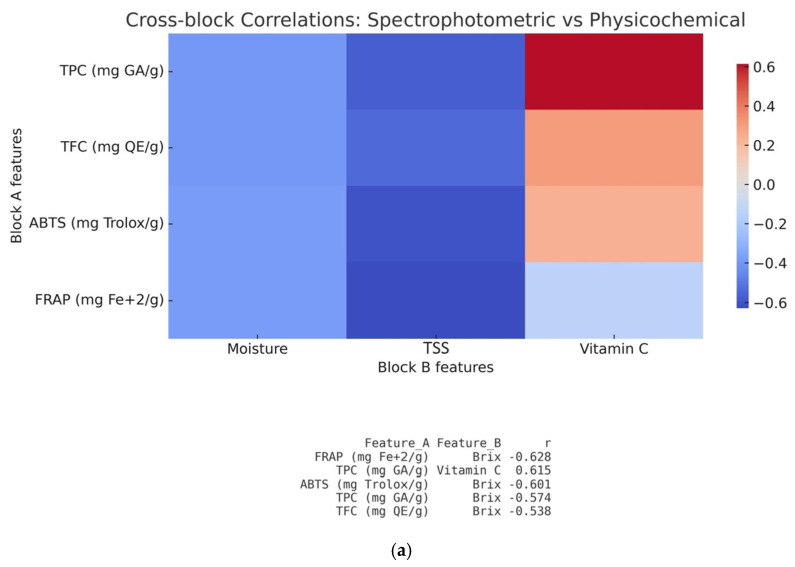

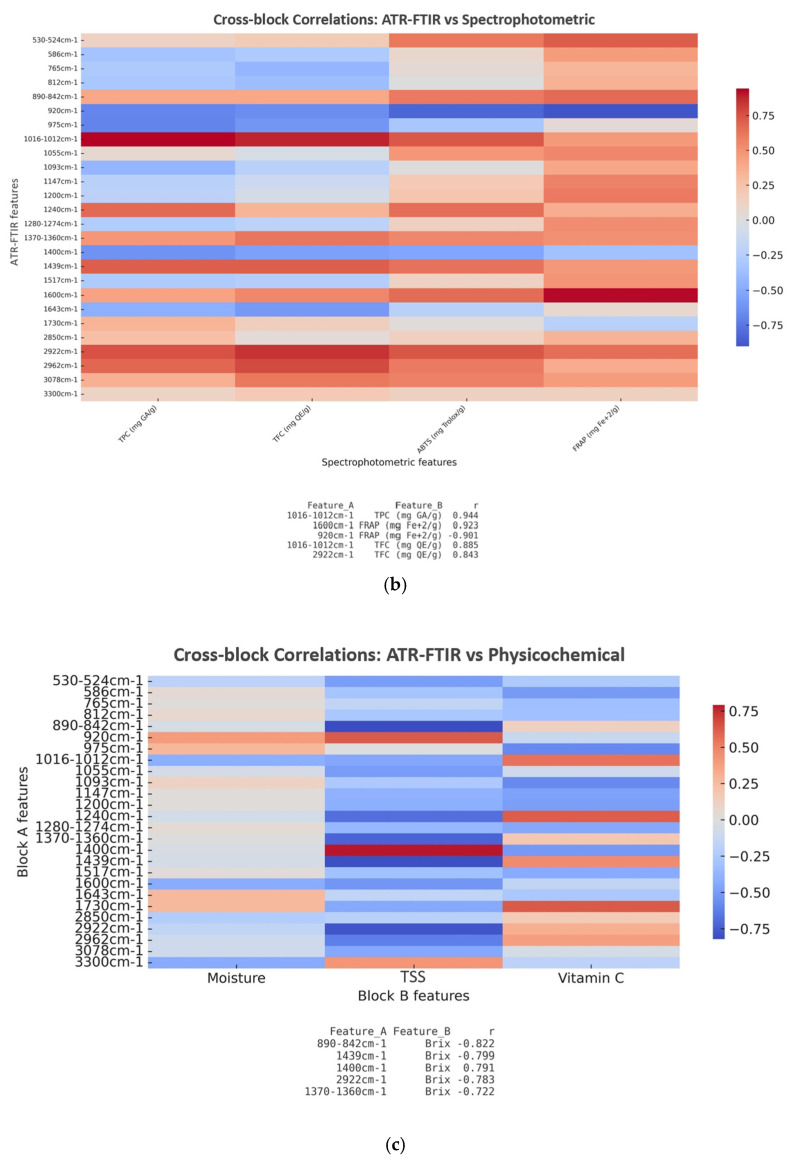
Cross-block score plot correlation matrices: (**a**) physicochemical and spectrophotometric, (**b**) ATR-FTIR and spectrophotometric, (**c**) ATR-FTIR and physicochemical.

As demonstrated in Figure 7a, the most notable correlations in the physicochemical and spectrophotometric models were observed between TPC and vitamin C (r = 0.615), as well as between FRAP and TSS (°Brix) (r = –0.628). This indicates that a higher phenolic content, and consequently antioxidant activity, is related to a greater Vitamin C content. This observation is consistent with the well-established antioxidant role of vitamin C, which explains its positive correlation with TPC [14,21,24,77,103] and could potentially suggest a synergistic contribution to the overall antioxidant potential of citrus peels. Moreover, not only FRAP but also TPC, TFC and ABTS^•+^ all showed negative correlations with TSS (°Brix), suggesting that samples richer in soluble solids (e.g., sugars) tended to exhibit a lower antioxidant activity. Similar results were reported by Dong et al. [18], who observed negative correlations among soluble solids, TPC and the antioxidant potency composite (APC) in lemon peels.

The cross-block correlation heatmap regarding the fusion of ATR-FTIR and spectrophotometric blocks (Figure 7b) reveals a strongly aligned association between ATR-FTIR spectra bands and antioxidant activity as it was captured by spectrophotometric assays. The most significant positive correlations were observed between the peak in the range 1016–1012 cm^−1^ and TPC (r = 0.944), the ATR-FTIR spectra band at 1600 cm^−1^ and FRAP (r = 0.923) and the ATR-FTIR spectra band at 2922 cm^−1^ and TFC (r = 0.843), while a strong negative correlation appeared between 920 cm^−1^ and FRAP (r = –0.901). The strong positive associations across these bands confirm that the molecular fingerprints captured by ATR-FTIR directly reflect the phenolic and flavonoid composition, confirming the aforementioned interpretation of the ATR-FTIR spectra.

Lastly, the fusion of ATR-FTIR and physicochemical block datasets (Figure 7c) displays mostly strong negative correlations between specific spectral regions and TSS (°Brix) values, while correlations with moisture and vitamin C were less pronounced. The most notable negative correlations were between TSS (°Brix) and ATR-FTIR bands at 2922 cm^1^, 1370–1360 cm^−1^, 1439 cm^−1^ and 1370–1360 cm^−1^, all associated with carbohydrates and the pectin structure [83,84,87,88], indicating that an increase in soluble solids is associated with a decrease in pectin-related absorbance. Pectin degradation contributes to tissue softening, as it becomes depolymerized and de-esterified, releasing soluble sugars [95]. Differences in pectin structure, such as the degree of esterification and side-chain composition [104], could likely explain the variation in TSS (°Brix) values and ATR-FTIR bands, revealing that pectin breakdown influences sugar accumulation and, consequently, peel composition. However, a more detailed investigation should be carried out.

Overall, the cross-block fusion confirms the crucial role of the ATR-FTIR block as the primary discriminant source and shows that the strongest synergy occurred between the physicochemical and spectrophotometric blocks. Combining these two datasets led to a stronger discriminative model compared with either single block. Even though their combined accuracy (0.83 ± 0.05) is lower, the synergy demonstrates that the two types of measurements enhance each other and reinforce the consistency between independent analytical techniques, which increases the scientific credibility. This strong synergy observed in the cross-block fusion suggests that multi-block integration could lead to a more comprehensive discrimination of citrus peel classes by exploiting their combined information.

#### 3.5.3. Multi-Block Fusion

**Figure 8:**
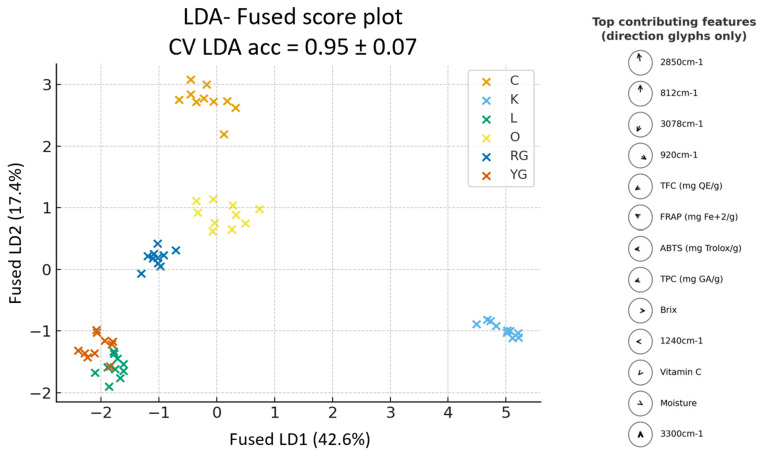
Multi-block fused score plot and the top contributing features in discrimination.

The fused score plot (Figure 8) shows a clear and well-defined class discrimination among all citrus peel classes across the two linear discriminants (LD1, LD2). The classes are well-clustered and evenly distributed across the discriminant space, indicating that the fusion of all blocks results in a balanced and synergistic model that efficiently captures both molecular and compositional variability.

The fusion of all three blocks enhances the interpretive strength by connecting molecular absorbance with the concentration of antioxidant compounds and physicochemical traits, leading to a consistent and comprehensive sample discrimination. The top contributing features show that polyphenolic variables, such as 2850 cm^−1^, 812 cm^−1^, 920 cm^−1^, TPC, TFC, FRAP and ABTS^•+^ [84,89,99], drive the separation and imply a possible synergistic interaction among the antioxidant compounds of citrus peels, including phenolic acids, flavonoids and essential oils [71,105]. Interestingly, the partial overlap between the yellow grapefruit (YG) and lemon (L) classes could suggest similar biochemical profiles, as this similarity likely could be associated with their related compositional and environmental traits, such s as similar level of polyphenols and antioxidant activity, as is evident from the TFC, TPC, ABTS, vitamin C content and the peak at 1240 cm^−1^.

Compared with the single-block models, where the discrimination was moderate and often overlapping, especially in the spectrophotometric and physicochemical blocks, the multi-block fusion yields a much stronger and clearer class discrimination. Compared to the cross-block fusions, which also demonstrated high accuracies, the multi-block model provides a similarly strong classification while offering an enhanced interpretability through the joint contribution of all features. The multi-block fusion confirms that, while the ATR-FTIR block carries the dominant discriminatory information, the spectrophotometric and physicochemical blocks contribute synergistically to a stronger, more stable and distinct class discrimination, enhancing the overall reliability of the multi-block analysis model than either single-block or cross-block models alone.

Moreover, the multi-block fusion score plot (Figure 8) not only presented a clear discrimination among citrus peel classes but also successfully distinguished samples originating from different geographical regions. In particular, lemon (L), orange (O), clementine (C), red grapefruit (RG) and yellow grapefruit (YG) peels from Greece formed well-defined clusters that were distinctly separated from the South African kumquat peels (K), reflecting possibly underlying differences in biochemical composition and physicochemical properties due to environmental and climatic factors. Interestingly, lemon (L) and yellow grapefruit (YG) peels, both cultivated in the same geographical area, Aigio, Achaia, Greece, exhibited a close proximity and minimal overlap within the discriminant space, consistent with their shared geographical and agro-environmental (climate, soil quality, temperature, humidity) background. The similarities between lemons and yellow grapefruit peels are also consistent with all textural features derived from the grayscale image analysis of the samples, according to which both samples showed an increased uniformity of their structure. Nevertheless, further investigation is necessary to clarify the environmental and biochemical factors responsible for the observed classes discrimination.

**Figure 9:**
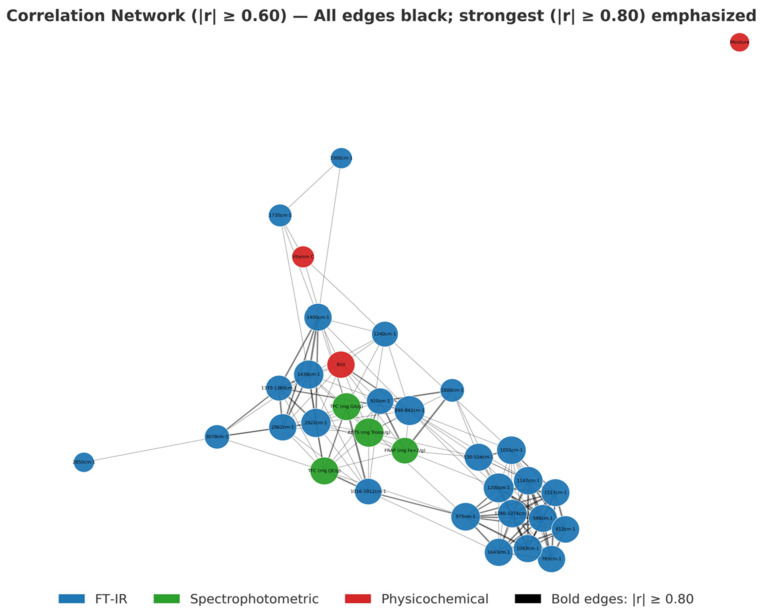
Multi-block correlation network (|r| ≥ 0.60; bold edges |r| ≥ 0.80); ATR-FTIR = blue, spectrophotometric = green, physicochemical = red).

Furthermore, a correlation network (Figure 9) using a Pearson correlation threshold of |r| ≥ 0.60 (with bold edges indicating the strongest associations, |r| ≥ 0.80) was developed, providing a visual representation of the interrelationships among the ATR-FTIR, spectrophotometric and physicochemical blocks. Notably, the correlation network highlights the role of the ATR-FTIR block as the primary fusion driver, as it is strongly connected to both the spectrophotometric and physicochemical datasets, which could explain why the ATR-FTIR-based cross-block score plots (Figure 6b,c) achieved the highest discrimination accuracies among the cross-block models. Furthermore, it reveals a more effective integration in the multi-block fusion, allowing the model not only to preserve the discriminative power of the ATR-FTIR block but also to enhance the model stability, interpretability and reliability of the spectrophotometric and physicochemical blocks.

A particularly strong cross-block correlation was observed between the 1016–1012 cm^−1^ peak, which suggests mostly the vibrations of carbohydrates, carboxylic acids and alcohols [88,91,93], and the total phenolic (TPC) and total flavonoid (TFC) content. The correlation with TPC could be attributed to the chemical composition of citrus peels, as they are rich in phenolic acids such as chlorogenic, caffeic, ferulic and p-coumaric acid [18,106,107], compounds that contribute significantly to TPC. Moreover, flavonoids, particularly flavanones glycosides (e.g., hesperidin, naringin, narirutin), which are abundant in citrus peels, may contribute weakly to this region through their glycosidic bond [24,71,108,109]. This correlation is further supported by the fact that citrus peels are also rich in cell wall polysaccharides (e.g., pectin, cellulose hemicellulose) [105,110]. According to Liu et al. [111], various structures of cell wall polysaccharides and polyphenols have been observed to exhibit characteristic interaction patterns, although the mechanisms are not yet fully understood, while Zhu [112] reported that noncovalent interactions between cell wall polysaccharides and polyphenols could influence the physicochemical properties of food matrices.

Additionally, a noteworthy correlation was identified between vitamin C and the ATR-FTIR bands at 1730 cm^−1^, which are related to organic acids such as ascorbic acid and ester functional groups [46,80], and at 1400 cm^−1^, which suggests the COOH symmetric stretching vibration of the pectin structure and the combination of O-H bending and C-H rocking in monosaccharides [46,83]. The correlation between vitamin C, 1400 cm^−1^ and 1730 cm^−1^ could suggest a shared chemical basis linked to carboxyl and carbonyl functionality. These results indicate that variations in the vitamin C concentration could possibly be associated with differences in the degree of pectin esterification and de-esterification in citrus peels, as a relationship between pectic enzyme activity and ascorbic acid content has been reported in plant tissues [113]. However, this is an active area of research and further investigation is needed.

Another strong correlation was observed among the FRAP assay and the ATR-FTIR bands at 920 cm^−1^, which is attributed to alkenes and phenolic compounds [84,86], and at 1600 cm^−1^, which is associated with pectin and aromatic compounds [83,91,92,93]. The peak at 920 cm^−1^ showed a strong negative correlation with the FRAP assay that could be attributed to different antioxidant mechanisms [21]. While the FRAP assay mainly quantifies hydrophilic, redox-active antioxidants [114], the spectra band at 920 cm^−1^ also captures nonpolar unsaturated components such as essential oils and their constituents (e.g., limonene, β-myrcene, pinene) [9,10,109] which exhibit a lipophilic character [115]. Therefore, samples with higher levels of essential oils may present strong peaks at 920 cm^−1^ but lower FRAP values, leading to the observed negative correlation. On the other hand, the strong positive correlation of the band at 1600 cm^−1^ confirms the strong antioxidant activity of citrus peels, which is primarily attributed to polyphenols [16,21,24,71], and also further supports and reinforces previously reported findings on the antioxidant activity of citrus peel pectin [104,116]. Altogether, this correlation highlights the strong antioxidant activity of citrus peels and the role of essential oils and their constituents in the overall antioxidant system, underscoring the need for more in-depth analyses.

To conclude, a multi-block model was employed to identify shared information across multiple datasets while discriminating among different classes [117]. The integration of information from multiple datasets reveals biochemical similarities, whereas other approaches (such as PCA) may inadvertently introduce class separation driven by noise or non-discriminative variance, rather than capturing the true class-related structure [118]. Previous studies in citrus fruits [59,119,120,121] have mainly applied single-block multivariate techniques, such as Principal Component Analysis (PCA), Hierarchical Cluster Analysis (HCA) and Partial Least Squares Regression (PLSR), to evaluate compositional or environmental parameters. However, these approaches remain limited to single-dataset analyses and cannot capture cross-correlations among diverse biochemical and compositional blocks. In contrast, the multi-block model used in this study offers a more comprehensive analysis, providing insight into the potential synergistic relationships between the different analytical domains and overcoming the limitations of conventional multivariate analyses.

## 4. Conclusions

Aiming to conduct a comprehensive comparative study of six different citrus peels, instrumental analytical techniques, along with an image texture analysis, were employed, leading to several noteworthy findings summarized below.

An analysis of grayscale image-derived textural features (mean intensity, standard deviation, contrast, dissimilarity, energy, homogeneity, correlation, angular second moment, short run emphasis, long run emphasis and run length non-uniformity) revealed that lemon and yellow grapefruit peels exhibit a greater structural uniformity, whereas clementine and red grapefruit peels are less uniform. Scientific reports indicate that factors such as climate and peel carotenoid content may contribute to these textural characteristics. Nevertheless, further research is needed to explore potential correlations with these or other factors.

Analyses of the physicochemical parameters provided critical insights on the quality attributes and stability of citrus peels, which may serve as potential indicators for their utilization in various applications. In particular, the high moisture content of citrus peels, ranging from 63,92% in orange peels to 74,95% in kumquat peels, can be described as a crucial factor for dehydration process design. Moreover, the significantly (*p* < 0.05) higher ascorbic acid (vitamin C) concentration in the peels of yellow and red grapefruit compared to the other peels, whose values did not differ significantly, further enhances the value of these by-products, as it is a compound known for its antioxidant activity and health-promoting effects.

Spectrophotometric assays also confirmed the high antioxidant and antiradical activity of citrus peels, particularly for red and yellow grapefruit and lemon. Notably, the similar variation pattern of antioxidant activity to that of total phenolic content (TPC) and total flavonoid content (TFC) confirmed the contribution of these compounds to the overall antioxidant capacity of the peels, as reported in the literature. These findings have important practical implications for the industrial valorization of citrus peels, as their high antioxidant and antiradical activity makes them a promising resource for developing nutraceuticals and natural additives.

Consistent with the spectrophotometric findings, ATR-FTIR spectra exhibited characteristic absorption bands corresponding to phenolic and aromatic compounds, as well as lipids and carbohydrates such as pectin and cellulose, which is in full agreement with the literature. Notably, citrus peels exhibiting a high carbohydrate content could be utilized for pectin extraction or biofuel production.

Moreover, the multi-block model integrated multiple datasets to uncover shared biochemical patterns and improve class discrimination, capturing cross-correlations across datasets and providing a more comprehensive understanding of the interactions among the analytical domains.

To sum up, the implementation of various destructive and non-destructive methods, combined with an increased sample size, provided valuable key insights and additional evidence regarding the chemical composition of citrus peels. Nonetheless, certain limitations remain and require further investigation. Future studies could focus on field-harvested citrus fruits to minimize the effects of post-harvest handling and storage, addressing the limitations of commercially sourced samples, as well as on additional citrus species and/or by-products, employing advanced statistical approaches. Furthermore, the comprehensive quantitative and qualitative characterization of phytochemicals and bioactive compounds, in addition to the use of in silico tools, could facilitate and contribute to the development of innovative products.

## Figures and Tables

**Table 1:** Moisture content (%), ascorbic acid content (mg/100 g DW) and total soluble solids (°Brix) of citrus peel samples.

Sample	Moisture Content (%) (Fresh Peel)	Ascorbic Acid (mg/100 g DW)	TSS (°Brix)
Yellow Grapefruit (*C. paradisi*)	67.76 ± 4.00 a	114.12 ± 12.71 a	10.73 ± 0.90 a
Red Grapefruit (*C. paradisi*)	69.85 ± 4.21 ab	104.54 ± 12.54 a	10.30 ± 1.34 a
Lemon (*C. limon*)	68.36 ± 5.66 ab	56.78 ± 12.64 b	10.00 ± 1.70 a
Orange (*C. sinensis* cv. Valencia)	63.92 ± 5.61 a	65.13 ± 12.69 b	15.55 ± 1.44 b
Clementine (*C. clementina*)	69.82 ± 5.62 ab	56.89 ± 12.67 b	10.80 ± 1.69 a
Kumquat (*C. margarita*)	74.95 ± 3.53 b	65.01 ± 5.07 b	17.43 ± 2.38 b

The results refer to the mean ± standard deviation. Mean values in the same column with different letters differ significantly (*p* < 0.05).

**Table 2:** Total phenolic content (TPC), total flavonoid content (TFC), antioxidant activity (FRAP) and antiradical activity (ABTS^•+^) of citrus fruit peels.

Sample	TPC (mg GAE/g DW)	TFC (mg QE/g DW)	FRAP (mg Fe^+2^/g DW)	ABTS^•+^ (mg Trolox/g DW)
Yellow Grapefruit (*C. paradisi*)	18.21 ± 0.56 a	27.01 ± 2.00 a	31.82 ± 2.16 a	19.92 ± 1.37 a
Red Grapefruit (*C. paradisi*)	17.42 ± 0.83 a	24.30 ± 1.83 a	30.32 ± 1.45 a	14.26 ± 0.65 b
Lemon (*C. limon*)	14.29 ± 1.27 b	31.90 ± 2.16 b	42.10 ± 2.12 b	18.66 ± 0.86 a
Orange (*C. sinensis* cv. Valencia)	13.62 ± 1.05 b	24.16 ± 2.13 a	34.84 ± 3.44 ac	16.13 ± 1.15 b
Clementine (*C. clementina*)	9.90 ± 0.72 c	13.91 ± 1.07 c	39.50 ± 2.62 bc	15.39 ± 1.12 b
Kumquat (*C. margarita*)	4.86 ± 0.53 d	7.63 ± 0.98 d	13.16 ± 0.98 d	6.71 ± 0.40 c

The results refer to the mean ± standard deviation. Mean values in the same column with different letters differ significantly (*p* < 0.05).

**Table 3:** Pearson correlations among total phenolic content (TPC), total flavonoid content (TFC), antiradical activity and antioxidant activity of citrus fruit peels.

Variables	TPC	TFC	Antiradical Activity	Antioxidant Activity
TPC	1	0.8592	0.5348	0.8097
TFC		1	0.6771	0.8532
Antiradical Activity			1	0.8389
Antioxidant Activity				1

Correlation is significant at the 0.05 level (2-tailed).

**Table 4:** The spectral intensities of citrus fruit peels.

Regions (cm^−1^)	Orange	Lemon	Red Grapefruit	Yellow Grapefruit	Clementine	Kumquat
3630	-	0.010 ± 0.002	-	-	-	-
3525	-	-	-	-	0.017 ± 0.003	-
3414	-	-	-	-	0.030 ± 0.006	-
3300	0.789 ± 0.005 a	0.049 ± 0.005 b	0.052 ± 0.004 b	0.071 ± 0.005 c	0.022 ± 0.003 d	0.810 ± 0.019 a
3078	-	0.064 ± 0.006 a	-	0.031 ± 0.001 b	-	-
3010	-	0.016 ± 0.003 a	0.021 ± 0.001 b	0.023 ± 0.002 b	-	-
2962	-	0.126 ± 0.015 a	0.077 ± 0.005 b	0.092 ± 0.004 c	-	-
2922	0.255 ± 0.024 a	0.483 ± 0.022 b	0.414 ± 0.031 c	0.388 ± 0.018 c	0.299 ± 0.016 d	0.168 ± 0.005 e
2850	0.085 ± 0.002 a	-	0.104 ± 0.001 b	0.034 ± 0.004 c	0.108 ± 0.010 b	-
1730	0.046 ± 0.006 a	0.116 ± 0.006 b	0.160 ± 0.019 c	0.149 ± 0.011 c	0.094 ± 0.007 d	0.119 ± 0.014 b
1643	-	0.046 ± 0.005 a	0.076 ± 0.003 b	0.055 ± 0.004 c	0.298 ± 0.011 d	0.118 ± 0.011 e
1600	0.237 ± 0.022 a	0.255 ± 0.024 a	0.199 ± 0.017 b	0.141 ± 0.007 c	0.283 ± 0.008 d	-
1517	0.024 ± 0.002 a	0.076 ± 0.005 b	0.018 ± 0.003 c	0.023 ± 0.004 ac	0.211 ± 0.007 d	0.023 ± 0.003 ac
1439	-	0.228 ± 0.022 a	0.200 ± 0.023 a	0.200 ± 0.021 a	0.088 ± 0.008 b	-
1400	0.064 ± 0.004 a	-	-	-	0.028 ± 0.002 b	0.049 ± 0.002 c
1370–1360	0.023 ± 0.003 a	0.093 ± 0.007 b	0.064 ± 0.004 c	0.064 ± 0.005 c	0.051 ± 0.003 d	0.032 ± 0.003 e
1330	-	0.013 ± 0.001 a	-	0.018 ± 0.002 b	-	-
1300	-	-	-	-	0.050 ± 0.005	-
1280–1274	-	0.049 ± 0.003 a	-	-	0.137 ± 0.004 b	-
1240	0.045 ± 0.003 a	0.064 ± 0.003 b	0.082 ± 0.003 c	0.105 ± 0.004 d	0.078 ± 0.007 c	0.040 ± 0.004 a
1200	-	0.052 ± 0.002 a	-	-	0.087 ± 0.006 b	-
1182	-	-	-	-	0.061 ± 0.002	-
1147	-	0.055 ± 0.008 a	-	-	0.109 ± 0.006 b	-
1093	-	0.081 ± 0.009 a	-	-	0.123 ± 0.005 b	0.034 ± 0.004 c
1055	-	0.044 ± 0.003 a	-	0.062 ± 0.005 b	0.096 ± 0.010 c	-
1016–1012	0.545 ± 0.042 a	0.538 ± 0.021 a	0.636 ± 0.026 b	0.610 ± 0.039 b	0.228 ± 0.009 c	0.117 ± 0.009 d
975	-	0.053 ± 0.003 a	-	-	0.142 ± 0.005 b	0.077 ± 0.005 c
920	0.019 ± 0.002 ab	0.018 ± 0.002 ab	0.021 ± 0.002 a	0.020 ± 0.005 a	0.015 ± 0.002 b	0.051 ± 0.004 c
890	0.016 ± 0.003 a	0.188 ± 0.016 b	0.126 ± 0.010 c	0.149 ± 0.031 c	-	0.016 ± 0.002 a
842	-	-	-	-	0.035 ± 0.001	-
812	0.017 ± 0.002 a	0.024 ± 0.002 b	0.024 ± 0.002 b	0.018 ± 0.003 a	0.102 ± 0.003 c	0.021 ± 0.002 ab
765	0.024 ± 0.004 a	0.014 ± 0.001 b	0.014 ± 0.001 b	0.022 ± 0.003 a	0.077 ± 0.004 c	0.018 ± 0.002 d
738	-	-	-	-	0.061 ± 0.004	-
669	-	-	-	-	0.015 ± 0.001	-
623	0.012 ± 0.001 a	-	-	0.015 ± 0.002 a	0.051 ± 0.002 b	-
586	0.008 ± 0.002 a	0.018 ± 0.002 b	0.007 ± 0.001 a	0.008 ± 0.001 a	0.035 ± 0.005 c	0.011 ± 0.001 d
530–524	0.016 ± 0.003 a	0.029 ± 0.004 bd	0.009 ± 0.001 c	0.024 ± 0.003 b	0.032 ± 0.005 d	0.009 ± 0.002 c

The results refer to the mean ± standard deviation. Mean values in the same row with different letters differ significantly (*p* < 0.05).

**Table 5:** Main spectral intensities of citrus fruit peels.

Regions (cm^−1^)	Band	Compounds
3630–3410	ν (OH)	Pectin, phenolic compounds
3300	ν (OH)	Water, carbohydrates (pectin and cellulose), organic acids, polyphenols
2962–2922	ν (C(sp^3^)-H)	Carbohydrates, carboxylic acids, flavonoid glycosides
2850		
1730	ν (C=O)	Organic acids, esters, cutin
1643	δ (OH)	Polyphenols, carbohydrates, organic acids, water
	ν (C=O)	Flavonoids
1600	ν (C−C)	Pectin, aromatic compounds
	ν (COOH)	Pectin
1517	ν (C=C-C) (aromatic)	Phenolic compounds
1439–1400	δ (C-H)	Carbohydrates (e.g., pectin)
	δ (O-H)	
	ν (COOH)	
1370–1360	ν (CH_3_)	Organic acids, carbohydrates
	ν (C-OOH)	
1330–1330	δ (C-H)	Polysaccharides (e.g., cellulose)
1280–1274	δ (O-H)	Cutin, polysaccharides
	ν (C-N)	Proteins (amide III band)
	δ (N-H)	
	δ (O=C-N)	
1240	ν (C-O)	Polyphenols, carbohydrates
	ν (C=C)	Aromatic polyphenols
1182	ν (C-O-C)	Cellulose
	ν (C-C)	
1147	ν (C-O-C)	Cutin, pectin, cellulose
1100–1090	ν (C-O)	Pectin, polysaccharides
	ν (C-C)	
1055	δ (C-O)	Carbohydrates (cellulose, sucrose)
	ν (C-OH)	
1016–1012	ν (C-O)	Pectin, cellulose
	ν (C-C)	
	δ (C-OH)	Carboxylic acids, alcohols, carbohydrates
	δ (O-CH)	Pectin, polysaccharides
975	δ (*trans* C-H)	Carotenoids
920	δ (C=C)	Alkenes
	δ (C-H)	Benzene ring of phenols
890–840	δ (C-H)	Para (1,4)-substituted aromatic rings
812	C-H	Aromatic ring of phenols
765	δ (OH)	C-OH group
	δ (C-H)	Ortho (1,2)-substituted aromatic rings
738	δ (*cis* C-H)	Carotenoids
623	δ (O-H)	Pectin
586	δ (C-H)	Polyphenols and flavonoids
530–524	δ (C-O-C)	Glycosidic bond of pectin

δ—bending vibration, ν—stretching vibration.

## Data Availability

The original contributions presented in this study are included in the article. Further inquiries can be directed to the corresponding author.
